# Association between Child Tax Credit advance payments and food insufficiency in households experiencing economic shocks

**DOI:** 10.1093/haschl/qxae011

**Published:** 2024-01-31

**Authors:** Nicole C McCann, Lorraine T Dean, Allison Bovell-Ammon, Stephanie Ettinger de Cuba, Tiffany Green, Paul R Shafer, Julia Raifman

**Affiliations:** Department of Health Law, Policy, and Management, Boston University School of Public Health, Boston, MA 02118, United States; Department of Epidemiology, Johns Hopkins Bloomberg School of Public Health, Baltimore, MD 21205, United States; Department of Pediatrics, Boston Medical Center, Boston, MA 02118, United States; Department of Pediatrics, Boston University Chobanian and Avedisian School of Medicine, Boston, MA 02118, United States; Department of Health Law, Policy, and Management, Boston University School of Public Health, Boston, MA 02118, United States; Department of Pediatrics, Boston University Chobanian and Avedisian School of Medicine, Boston, MA 02118, United States; Departments of Population Health Sciences and Obstetrics and Gynecology, University of Wisconsin-Madison, Madison, WI 53706, United States; Department of Health Law, Policy, and Management, Boston University School of Public Health, Boston, MA 02118, United States; Department of Health Law, Policy, and Management, Boston University School of Public Health, Boston, MA 02118, United States

**Keywords:** advance child tax credit, insecurity, food], policies, social, family health

## Abstract

The COVID-19 pandemic brought increases in economic shocks due to poor health and lost employment, which reduced economic well-being, especially in households with children. The American Rescue Plan Act of 2021 expanded Child Tax Credit (CTC) payments to include eligibility for the lowest income households, boosted benefit levels, and provided monthly advance payments to households with children. Using Census Household Pulse Survey respondent data from January 2021 to July 2022, we evaluated the association between these advance CTC monthly payments and food insufficiency among households with children experiencing health- or employment-related economic shocks (defined as missed work due to COVID-19/other illness or COVID-19–related employer closure/layoff/furlough). Using a triple difference design, we found that the advance CTC was associated with greater reductions in food insufficiency among households with children experiencing economic shocks both compared with households without children and with households with children not experiencing economic shocks. Permanently expanding the advance CTC could create resilience to economic shocks during disease outbreaks, climate disasters, and recessions.

## Introduction

Many families in the United States live paycheck to paycheck, making them vulnerable to unexpected economic shocks, such as illness, hospitalization, and job loss.^1-3^ Such shocks can lead to hardships, including deepened poverty and food insufficiency.^1-4^ This is of concern for families with children, as even brief periods of food insufficiency can be detrimental to child health, development, and education.^5-10^ Families with children are more likely to experience poverty and economic shocks, and Black and Hispanic families are particularly vulnerable due to discrimination and structural racism, which have driven income and wealth disparities.^11-14^ Thus, policies that can address food insufficiency among populations vulnerable to economic shocks are key for achieving health equity.

The COVID-19 pandemic brought widespread economic shocks across the United States, with more than 50 million people losing employment. Additionally, missing work due to illness increased by 50% compared with the 2 years prior.^15,16^ Negative effects were disproportionately concentrated among low-income, Black, Hispanic, and/or families with children who were most likely to miss work and less likely to receive paid sick leave.^15^ Further, these groups were also more likely to experience increases in food insufficiency.^12,13^

To mitigate negative economic consequences of COVID-19, Congress passed the American Rescue Plan Act (ARPA) in March 2021. The ARPA included 3 modifications to the existing Child Tax Credit (CTC): (1) eligibility for the full CTC amount was expanded to families with low/no income; (2) the maximum credit amount increased from $2000 to $3000 per qualifying child for children aged 6–17 years (previously ended at 16 years) and to $3600 for children aged 5 years or younger, with greater gains for low-income households; and (3) payments were delivered as monthly per-child advances of $200–$300 during the 6-month period from July 2021 through December 2021. Families received the rest of the increased CTC amount for 2021 when filing taxes in 2022. In early 2022, the CTC expansion expired and reverted to its original structure, with lower credit amounts, no monthly advance payments, and non-refundability, excluding those with the lowest incomes.

Families reported using advance CTC monthly payments for necessities such as food and clothing, and payments did not lead to decreased labor supply among recipients.^17,18^ The advance CTC was associated with decreased food insufficiency among households with children during its implementation and with a corresponding increase in food insufficiency after its expiration.^19-22^ However, it is still unclear if the advance CTC had differential impacts for vulnerable households, such as those experiencing economic shocks.

The objective of our study was to investigate how the ARPA expansion of the CTC, particularly the advance payments, impacted food insufficiency, including how it may have differentially protected households vulnerable to COVID-19–driven economic shocks. We aim to expand on prior literature to understand how the advance CTC may differentially affect vulnerable households and why it may have differential effects across demographic groups, including how the payments may support households to maintain food sufficiency and increase resilience to economic shocks in periods such as disease outbreaks, climate disasters, or recessions.

## Data and methods

### Sample and study period

We used the US Census Bureau's nationally representative Household Pulse Survey (HPS), which contains respondent self-reported demographics, household composition, employment, and economic hardship data (including food insufficiency and participation in various assistance programs).^23^ In HPS, only 1 adult responds on behalf of the household. We limited the sample to adults younger than 65 years to capture those of working age with primarily their own children in the household, and to individuals without missing data (except for income, for which a missing category was created).^19,20^ We included data from HPS waves 22–27, capturing 3 time periods: (1) before (January 6, 2021–July 5, 2021), (2) during (July 21, 2021–January 10, 2022), and (3) after (January 26, 2022–July 11, 2022) advance CTC implementation. These survey waves were selected based on the advance CTC payment dates of July 15, 2021, through December 15, 2021.

### Exposure: advance CTC payments

Through the ARPA in 2021, over 90% of families with children were eligible to receive $200–$300 monthly per child between July and December 2021. Families who received monthly payments were eligible to claim an additional $1500–$1800 per child after filing their 2021 tax return in early 2022.^19,24,25^

The advance CTC payment exposure was defined as living in a household with at least 1 child present during the survey period that covered months when the advance CTC payments were being dispersed (July 21, 2021–January 10, 2022).

### Exposure: economic shocks

We defined economic shocks as a report of at least 1 health-related shock or employment-related shock. Health-related shocks were defined as a report of missing work in the past 7 days for 1 of the following reasons: “I am/was sick with coronavirus symptoms or caring for someone sick with coronavirus symptoms” or “I am/was sick (not coronavirus-related) or disabled.” Employment-related shocks were defined as a report of missing work in the past 7 days for 1 of the following reasons: “I am/was laid off or furloughed due to the coronavirus pandemic,” or “my employer closed temporarily due to the coronavirus pandemic,” or “my employer went out of business due to the coronavirus pandemic.”

### Outcome

The outcome was household food insufficiency, defined as a binary measure. Following US Department of Agriculture (USDA) standard methods for coding survey responses,^26^ food insufficiency was defined as a report of “sometimes not enough to eat” or “often not enough to eat” in response to the following question: “In the last seven days, which of these statements best describes the food eaten in your household?” Respondents were not considered to have food insufficiency if they reported “enough of the kinds of food I/we wanted to eat” or “enough, but not always the kinds of food (I/we) wanted to eat” in response to this question.^23,26^

The HPS food-insufficiency measure of not having enough food to eat in the past 7 days is related to food insecurity, which is more expansive and based on a scale developed by the USDA. Food insufficiency is a narrower definition that focuses on food-intake quantity.^26^ Researchers used HPS data on food insufficiency to provide real-time information throughout the pandemic; several studies have used the HPS measure to gain insight into food access during the COVID-19 pandemic.^12,19,20,27,28^

### Covariates

Other relevant policy changes during the study period included the third and final Economic Impact Payment (EIP) in March 2021, the cessation of unemployment insurance expansions in June–September 2021, and the expiration of the federal eviction moratorium in August 2021. We adjusted for self-reported individual receipt of the EIP and/or unemployment insurance and eviction risk (somewhat/very likely) to account for these changes. We included covariates for participation in other public assistance benefits, including the Supplemental Nutrition Assistance Program (SNAP) and other food aid based on yes/no questions, as well as respondent health insurance coverage. We further adjusted for demographic characteristics, including sex assigned at birth, age group, educational level, prior-year household income, marital status, number of adults in the household, and number of children in the household. We also included survey wave and state fixed effects, which capture national trends that affected both households with children and households without children during each time period (eg, inflation) as well as time-invariant state characteristics not captured by other covariates.

### Analyses

We reported the unadjusted prevalence of any economic shocks, health-related shocks, and employment-related shocks prior to advance CTC implementation for all households, households with and without children, and stratified by subgroup (by race/ethnicity and for low-income households [earning <$35 000 in the prior year^20,21^]).

We also reported the unadjusted prevalence of household food insufficiency before, during, and after advance CTC implementation for (1) all households, (2) households without children not experiencing economic shocks, (3) households with children not experiencing economic shocks, (4) households without children experiencing economic shocks, and (5) households with children experiencing economic shocks, and by racial/ethnic and income subgroup.

We conducted linear regressions to evaluate the association between experiencing any economic shocks, health-related shocks, and employment-related shocks and household food insufficiency in the period prior to advance CTC implementation for all households, households with and without children, and by racial/ethnic and income subgroup. We adjusted for wave and state fixed effects, time-varying covariates including receipt of EIP, SNAP, and demographic characteristics (detailed above).

In our primary model, we conducted a difference-in-difference-in-differences (“triple difference”) analysis of household food insufficiency. We compared changes in household food insufficiency in the period during advance CTC implementation in households with children experiencing economic shocks relative to households without children and to households not experiencing economic shocks. The main exposure was a binary indicator for being in a household with children, interacted with a binary indicator for the period during advance CTC implementation, and with a binary indicator for households experiencing economic shocks. The resulting estimates speak to the association between the advance CTC and food insufficiency in households with children compared with those without children, and if this association varied depending on whether households were experiencing economic shocks. We used linear models and adjusted for state and wave fixed effects, demographics, and time-varying covariates described above (Appendix Methods). Triple difference relies on the parallel trends assumption, which assumes that underlying trends between households with and without children would be parallel in the absence of the advance CTC. To test this assumption, we evaluated whether differences in food-insufficiency trends varied over time between households with and without children in the period prior to advance CTC implementation. We tested this association using both a triple difference estimate (ie, interacting an indicator for the presence of children with an indicator for the presence of economic shocks with a continuous time indicator) and difference-in-difference estimates separately among households (1) experiencing and (2) not experiencing economic shocks (ie, interacting an indicator for the presence of children with a continuous time indicator in both models). We found that, before advance CTC implementation, there were no significant differences in food-insufficiency trends over time in naive and adjusted versions of all 3 models (Table S1).

To test the robustness of our findings, we clustered standard errors by state and varied inclusion of fixed effects. We also conducted 2 additional difference-in-difference analyses. We evaluated the interaction between advance CTC implementation and children in the household by estimating stratified models separately among households experiencing and not experiencing shocks. Additionally, some respondents may have received lump-sum tax refunds during the period after the advance CTC monthly payment expiration, depending on when they filed their tax return. Therefore, we conducted a sensitivity analysis in which we excluded dates after the tax filing deadline in April 2022 based on evidence that most Americans file their taxes close to the deadline.^29^ Last, we conducted the triple differences analysis limited to the low-income subgroup only, because this group was most likely to receive the greatest benefit from changes to the CTC made in the ARPA.^21,22^

This study was exempt from Institutional Review Board (IRB) approval and informed consent and meets Strengthening the Reporting of Observational Studies in Epidemiology (STROBE) reporting guidelines for cross-sectional studies.^30^ In all models, we used US Census–provided weights divided by the number of survey waves. We used 2-sided *t* tests or chi-square tests to test for significant differences between groups; *P* < .05 was considered significant. Analyses were conducted April through October 2023 using Stata/MP version 17.0 (StataCorp, College Station, TX).

## Results

### Sample characteristics

Our sample comprised 1 125 299 respondents, representing a weighted population of 122 800 808 individuals. Respondents were majority female (*n* = 691 775 [51.3%]) and non-Hispanic White (*n* = 808 207 [62.2%]), with a plurality (*n* = 463 735 [48.4%]) aged 25–44 years. Weighted individual demographics and household socioeconomic characteristics are shown in Table 1 for the full sample and stratified by household presence of children and experience of economic shocks.

**Table 1.** Sample characteristics, overall and for households experiencing or not experiencing economic shocks, and with or without children.

		Households not experiencing shocks (*n* = 1 041 692)			Households experiencing shocks (*n* = 84 607)			
Characteristic	Overall (*n* = 1 126 299)	Households without children (*n* = 602 659)	Households with children (*n* = 439 033)	*P* ^a^	Households without children (*n* = 52 924)	Households with children (*n* = 31 683)	*P* ^a^	*P* ^b^
Sex at birth								
Female	691 775 (51.3)	357 798 (48.1)	279 009 (55.2)	<.001	32 016 (49.2)	22 060 (58.7)	<.001	<.001
Male	434 524 (48.7)	244 961 (51.9)	160 024 (44.8)		20 016 (50.8)	9623 (41.3)		
Age group, y								
18–24	37 113 (7.4)	24 835 (8.8)	9831 (5.8)	<.001	1611 (6.3)	836 (5.3)	<.001	<.001
25–44	463 735 (48.4)	190 920 (39.5)	244 429 (61.7)		12 774 (32.2)	15 612 (58.0)		
45–64	625 451 (44.2)	386 904 (51.7)	184 773 (32.5)		38 539 (61.6)	15 235 (36.8)		
Race and ethnicity								
Hispanic	119 439 (16.2)	53 462 (13.0)	53 344 (19.1)	<.001	6310 (17.4)	6323 (27.4)	<.001	<.001
Non-Hispanic								
Asian	64 661 (5.3)	32 230 (5.2)	29 001 (6.0)	<.001	1939 (3.4)	1491 (3.7)	.101	<.001
Black	87 960 (12.3)	41 753 (10.5)	35 867 (13.3)	<.001	5401 (15.2)	4939 (22.1)	<.001	<.001
White	808 207 (62.2)	452 921 (67.7)	301 990 (57.6)	<.001	36 324 (59.1)	16 962 (42.2)	<.001	<.001
Another race or ethnicity	46 032 (3.9)	22 283 (3.6)	18 831 (4.0)	<.001	2950 (5.0)	1968 (4.6)	.075	<.001
Education								
Less than high school	23 189 (6.8)	8069 (4.6)	10 658 (8.2)	<.001	2157 (10.8)	2305(17.1)	<.001	<.001
High school/equivalent	121 961 (27.4)	61 511 (26.2)	43 930 (26.3)		9861 (38.3)	6659(39.1)		
Some college/2-y degree	350 892 (30.8)	185 664 (30.8)	128 416 (30.2)		23 162 (33.7)	13 650 (31.1)		
4-y degree or higher	630 257 (35.0)	347 415 (38.5)	256 029 (35.3)		17 744 (17.3)	9069 (12.7)		
Marital status								
Married	651 530 (50.5)	295 135 (41.9)	320 199 (64.8)	<.001	19 856 (32.7)	16 340 (46.7)	<.001	<.001
Not married	474 769 (49.5)	307 524 (58.1)	118 834 (35.2)		33 068 (67.3)	15 343 (53.3)		
Health insurance coverage								
Uninsured	143 494 (18.0)	71 214 (16.3)	54 558 (18.0)	<.001	10 647 (25.3)	7075 (29.0)	<.001	<.001
Public	105 078 (11.9)	41 206 (8.2)	36 715 (11.8)		17 762 (32.6)	9928 (31.2)		
Private	877 727 (70.1)	49 183 (75.6)	347 760 (70.2)		24 515 (42.1)	14 680 (39.7)		
Respondent employed in last 7 d	832 317 (69.5)	478 257 (77.6)	354 060 (76.2)	<.001	– (0)	– (0)	NA	<.001
Report of UI benefits as spending source in last 7 d	82 160 (8.6)	33 336 (6.3)	25 898 (7.2)	<.001	14 036 (25.6)	8890 (27.3)	.003	<.001
Current participation in SNAP in last 7 d by anyone in household	97 801 (13.1)	29 175 (6.9)	45 220 (16.5)	<.001	12 773 (27.2)	10 633 (38.7)	<.001	<.001
Receipt of food aid in last 7 d by anyone in household	59 485 (6.9)	18 499 (4.1)	30 507 (8.9)	<.001	5260 (11.0)	5219 (18.3)	<.001	<.001
Report of EIP as spending source in last 7 d	181 970 (18.8)	81 513 (15.7)	77 740 (20.8)	<.001	13 396 (25.6)	9321 (29.6)	<.001	<.001
Report of risk for eviction in next 2 mo	15 400 (2.3)	4846 (1.3)	6020 (2.4)	<.001	2370 (6.1)	2164 (8.9)	<.001	<.001
No. of adults in household								
1	229 091 (21.2)	149 631 (25.2)	57 154 (14.4)	<.001	15 796 (30.3)	6510 (21.5)	<.001	<.001
2	618 640 (52.0)	306 611 (48.4)	274 048 (58.9)		23 199 (41.7)	14 782 (45.1)		
3+	278 568 (26.9)	146 417 (26.4)	107 831 (26.6)		13 929 (28.0)	10 391 (33.4)		
No. of children in household								
0	655 583 (57.1)	602 659 (100)	0 (0)	<.001	52 924 (100)	0 (0)	<.001	<.001
1	204 083 (18.6)	0 (0)	188 793 (43.2)		0 (0)	15 290 (45.2)		
2	172 977 (15.1)	0 (0)	163 109 (35.6)		0 (0)	9868 (31.1)		
3+	93 656 (9.2)	0 (0)	87 131 (21.2)		0 (0)	6525 (23.7)		
Annual household income								
<$25 000	103 151 (13.5)	52 070 (12.3)	27 468 (10.5)	<.001	16 093 (34.7)	7520 (28.7)	<.001	<.001
$25 000–$34 000	76 070 (9.2)	41 346 (9.1)	24 359 (8.2)		6355 (13.0)	4010 (13.8)		
$35 000–$49 999	94 885 (10.0)	54 385 (10.6)	31 274 (9.0)		5768 (10.9)	3458 (10.5)		
$50 000–$74 000	156 514 (14.1)	91 894 (15.5)	53 751 (13.1)		6912 (11.4)	3957 (10.6)		
$75 000–$149 000	322 917 (23.6)	177 793 (25.1)	131 815 (24.7)		8352 (12.0)	4957 (10.9)		
$150 000+	218 736 (12.9)	112 136 (13.0)	101 773 (15.3)		2934 (3.3)	1893 (3.3)		
Missing	154 026 (16.7)	73 035 (14.5)	68 593 (19.3)		6510 (14.7)	5888 (22.2)		

Abbreviations: EIP, Economic Impact Payment; SNAP, Supplemental Nutrition Assistance Program; UI, unemployment insurance; NA, not applicable.

*n* = 1 126 999. Data are presented as *n* (weighted %). Values may not add up to 100% due to rounding. Source: Authors' analysis of Household Pulse Survey data from the Census Bureau, January 2021 to July 2022.

^a^*P* values within the “households not experiencing shocks” and “households experiencing shocks” columns represent the difference between households without and with children within each category, respectively.

^b^*P* values on the far-right column represent the difference between households experiencing and not experiencing shocks.

### Unadjusted household prevalence of economic shocks

The prevalence of households experiencing economic shocks in the period before advance CTC implementation was 10.9% (95% CI: 10.7%–11.0%), with 4.5% (95% CI: 4.4%–4.6%) reporting health-related shocks and 6.4% (95% CI: 6.2%–6.5%) reporting employment-related shocks (Table S2). Among all households, non-Hispanic Black, Hispanic, and low-income households were most impacted by economic shocks, with 15.5% (95% CI: 14.8%–16.1%), 14.3% (95% CI: 13.8%–14.9%), and 20.6% (95% CI: 20.1%–21.1%) experiencing economic shocks, respectively, compared with 9.2% (95% CI: 9.0%–9.3%) of White households and 8.2% (95% CI: 8.0%–8.4%) of higher-income households (Table S2, Figure S1).

### Unadjusted household food insufficiency prevalence before, during, and after advance CTC implementation

**Figure 1.**
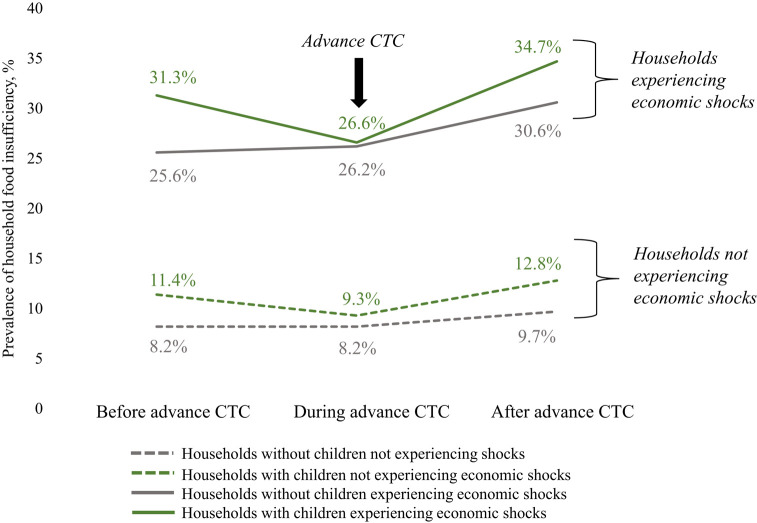
Unadjusted prevalence of household food insufficiency among households with and without children experiencing and not experiencing economic shocks before, during, and after advance Child Tax Credit (CTC) implementation. The figure shows the unadjusted prevalence of household food insufficiency in the periods before advance CTC implementation (January 6, 2021, through July 5, 2021), during advance CTC implementation (July 21, 2021, through January 10, 2022), and after advance CTC implementation (January 26, 2022, through July 11, 2022). The solid green line represents households with children experiencing economic shocks (health-related or employment-related shocks). The solid gray line represents households without children experiencing economic shocks. The green and gray dotted lines represent households with and without children, respectively, not experiencing economic shocks. Prevalence values are shown above or below each line. The dip in food insufficiency during the period of advance CTC implementation seen among households with children experiencing economic shocks is indicated with an arrow. Source: Authors' analysis of Household Pulse Survey data from the US Census Bureau, January 2021 to July 2022.

**Figure 2.**
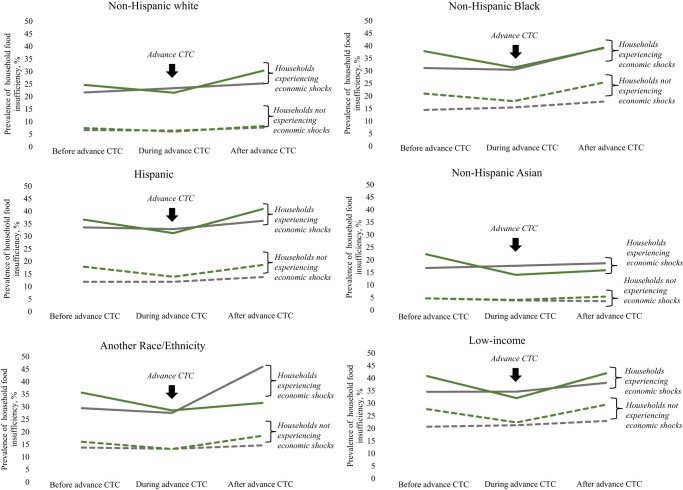
Unadjusted prevalence of household food insufficiency among households with and without children experiencing and not experiencing economic shocks before, during, and after advance Child Tax Credit (CTC) implementation: stratified by race, ethnicity, and low-income subgroups. The figure shows the unadjusted prevalence of food insufficiency in the periods before advance CTC implementation (January 6, 2021, through July 5, 2021), during advance CTC implementation (July 21, 2021, through January 10, 2022), and after advance CTC implementation (January 26, 2022, through July 11, 2022), stratified by race, ethnicity, and low-income subgroups. The solid green lines represent households with children experiencing economic shocks (health-related or employment-related shocks). The solid gray lines represent households without children experiencing economic shocks. The green and gray dotted lines represent households with and without children, respectively, not experiencing economic shocks. Dips in food insufficiency during the period of advance CTC implementation seen among households with children experiencing economic shocks are indicated with an arrow. A full table of unadjusted household food-insufficiency prevalence values is available in Table S3. Source: Authors' analysis of Household Pulse Survey data from the Census Bureau, January 2021 to July 2022.

As depicted in Figure 1, unadjusted household food-insufficiency prevalence was elevated among households experiencing economic shocks (solid lines) relative to households not experiencing economic shocks (dashed lines). In both circumstances, food insufficiency was higher in households with children (green lines) relative to households without children (gray lines). While food insufficiency increased over the study period, there was a temporary dip in food insufficiency in households with children during the period of advance CTC implementation. Overall, Hispanic, non-Hispanic Black, and low-income subgroups had the highest unadjusted household food insufficiency. Household food-insufficiency prevalence by race/ethnicity and low-income subgroups is shown in Figure 2 and Table S3. Except for Asian and “another racial category” respondents, the presence of children in the household was associated with similar or higher rebound rates of food insufficiency post–advance CTC expiration.

### Association between economic shocks and household food insufficiency

In regression analysis, after adjusting for fixed effects and all covariates, economic shocks were associated with a 7.7 percentage point (95% CI: 6.7–8.6; *P* < .001) higher probability of food insufficiency compared with households without shocks before implementation of the advance CTC—an 80% increase. This difference was 6.7 percentage points (95% CI: 5.6–7.8; *P* < .001) among households without children and 8.5 percentage points (95% CI: 7.0–10.0; *P* < .001) among households with children (Table S4).

### Difference-in-difference analyses: estimated association between advance CTC implementation and food insufficiency among households with children experiencing economic shocks

Our triple difference model estimated a 3.5 percentage point decrease (95% CI: 6.1–0.90 percentage points; *P* = .008) in the proportion of respondents who reported experiencing food insufficiency among households with children experiencing economic shocks compared with households without children and with those not experiencing shocks during the period of advance CTC implementation (Table 2, Table S5). This represents an 11% decrease compared with the period before advance CTC implementation. Results were robust to clustered standard errors and varied inclusion of fixed effects (Table S6).

**Table 2.** Change in household food insufficiency during implementation of advance CTC in households with and without children experiencing and not experiencing economic shocks.

	Household food insufficiency in households without children (*n* = 655 583), % (95% CI)			Household food insufficiency in households with children (*n* = 470 671), % (95% CI)			Triple difference estimate^a^	
	Before advance CTC	During advance CTC	After advance CTC	Before advance CTC	During advance CTC	After advance CTC	Change in household food insufficiency, percentage points (95% CI)	*P*
Households not experiencing shocks (*n* = 1 041 692)	8.2 (8.0, 8.5)	8.2 (8.0, 8.5)	9.7 (9.3, 10.0)	11.4 (11.1, 11.7)	9.3 (9.0, 9.7)	12.8 (12.4, 13.3)	—	
Households experiencing shocks (*n* = 84 562)	25.6 (24.6, 26.6)	26.2 (24.7, 27.6)	30.6 (28.6, 32.5)	31.3 (30.0, 32.7)	26.6 (24.9, 28.4)	34.7 (32.4, 37.1)	−3.5 (−6.1, −0.90)	.008

Abbreviation: CTC, Child Tax Credit.

Source: Authors' analysis of Household Pulse Survey data from the Census Bureau, January 2021 to July 2022.

^a^In triple difference analysis, comparison is between households with children and without children and between households experiencing shocks and not experiencing shocks.

Additionally, difference-in-difference models conducted separately among households experiencing and not experiencing economic shocks were consistent with triple difference results. In both groups, the advance CTC was associated with decreased household food insufficiency, but the decrease was larger for households experiencing economic shocks compared with households not experiencing shocks (5.2 and 1.6 percentage point decrease, respectively) (Table S7).

Our results held when we excluded the period after the 2022 tax filing deadline from the triple difference model, although the effect was slightly greater. When this period was excluded, there was a 3.9 percentage point decrease in the proportion of respondents reporting food insufficiency among households with children experiencing economic shocks compared with households without children and those not experiencing economic shocks (compared with a 3.5 percentage point decrease in the main analysis) (Table S8). When the triple difference analysis was limited to the low-income subgroup, the magnitude of the effect remained the same (a 3.5 percentage point decrease), but the effect was no longer significant, likely due to a large reduction (84% decrease) in sample size and resulting lack of precision (Table S9).

## Discussion

We found that the advance CTC was associated with a 3.5 percentage point and 11% differential decrease in the probability of experiencing food insufficiency for households with children experiencing economic shocks compared with households without children and households not experiencing economic shocks. This finding is consistent with prior work documenting a decrease in food insufficiency during advance CTC implementation and an increase after advance CTC expiration among all households with children.^19-21^ We additionally found that the advance CTC was associated with a greater reduction in food insufficiency among households with children experiencing economic shocks (ie, missing work due to sickness with COVID-19/other illness or employer closures/layoffs/furloughs) than among those not experiencing economic shocks. These results help to understand the mechanisms through which the advance CTC can protect against food insufficiency, through differentially impacting demographic groups most structurally vulnerable to economic shocks, including low-income, Hispanic, and Black populations. Such findings are relevant for federal and state policymakers, as Congress could consider restoration of the advance CTC and as state lawmakers consider similar models.^31,32^ These findings may also be relevant to broadly reducing vulnerability to food insufficiency in households with children, especially in preparation for events such as disease outbreaks, climate disasters, or recessions, which could produce economic shocks.

Our results parallel prior observational and quasi-experimental studies that have found that, both before and during the COVID-19 pandemic, unexpected events impacting employment contribute to economic hardship.^1^ For example, 1 study found a positive association between parental decline in employment and household food insecurity.^2^ Additional state and national survey data indicate that people who lost or missed work due to COVID-19 were most likely to report worse food access or greater food insufficiency.^3,33,34^ However, to our knowledge, our study is the first to evaluate how the relationship between the advance CTC and household food insufficiency varied by contemporaneous economic shocks experienced by household members.

Even before the COVID-19 pandemic, the United States had a higher rate of children living in poverty than most other Organization for Economic Cooperation and Development (OECD) countries, with disparities by race and ethnicity.^11,35^ These disparities are shaped by structural racism, including historical policies, such as slavery and redlining, and modern-day policies, such as low federal minimum wage, which have created inequities in education, income, and wealth.^36-38^ People with low income and wealth are vulnerable to food insufficiency if they face sudden health or employment shocks.^39^ This vulnerability had a particularly negative impact during the COVID-19 pandemic, when absences from work due to illness, child care, or other personal obligations increased by 50% compared with the 2 years prior, and low-income, non-Hispanic Black, and Hispanic employees were least likely to have paid sick leave.^15^

Consistent with prior findings, we observed similar socioeconomic and racial/ethnic disparities: during the COVID-19 pandemic, low-income, Black, and Hispanic populations disproportionately experienced economic shocks. In the period before advance CTC implementation, low-income, non-Hispanic Black, and Hispanic households were 151%, 68%, and 55% more likely to experience economic shocks compared with higher-income and White households, respectively. Similarly, we found that the prevalence of household food insufficiency was higher among low-income, non-Hispanic Black, and Hispanic households compared with higher-income White households over the study period. Our results suggest that these findings are related; experiencing economic shocks was associated with an 80% increase in household food insufficiency. In combination, our findings show that the advance CTC was associated with an especially protective impact on households experiencing economic shocks, which were disproportionately non-Hispanic Black, Hispanic, and low-income, suggesting that the advance CTC may be impactful in these racial, ethnic, and income groups. While prior work has described differential impacts of the advance CTC on food insufficiency across demographic groups,^20-22^ our findings provide insight into how differential effects may arise: through protecting structurally vulnerable households (including those most likely to have low wealth/income and unpaid sick leave) in times of missed work due to sickness or job loss.

Our study has limitations. First, the HPS relies on self-reported, repeated cross-sectional data, with low overall response rates. Because the data are repeated cross-sectional surveys rather than longitudinal, we could only evaluate contemporaneous associations between economic shocks and food insufficiency. Second, we defined economic shocks based on available survey response options and could not capture individuals who experienced economic shocks for other reasons. Third, respondents may have received lump-sum tax refunds during the period after the advance CTC monthly payment expiration. In fact, we found that, when we excluded dates after the tax filing deadline from analysis, the effect size was slightly greater, suggesting that we may be underestimating the impact of the monthly payments on food insufficiency in the absence of lump-sum returns. However, the difference was not large, which may be consistent with prior evidence that lump-sum and monthly payments may be used for different purposes (lump-sum payments more often used for arrears and monthly payments for ongoing costs).^40^ Fourth, we estimated the effect of the advance CTC on households eligible for the advance CTC (ie, households with children), rather than households that received the advance CTC. We used this “intent-to-treat” approach to avoid selection bias, as households that filed taxes during the advance CTC may differ from those that do not. This is a conservative approach that may bias results towards the null; the effect size may be greater only among households that received the advance CTC. Another limitation is that there may have been time-varying differences between households with and without children, such as school closures, which could have impacted food insufficiency. Finally, our triple difference analysis was underpowered when restricted to low-income households only (a subgroup highly impacted by the ARPA changes to the CTC); however, while we did not observe significant effects in this subgroup, the magnitude of the effect size was the same as in our main analysis, despite the loss of precision. Because of the proxy identification of benefits from the advance CTC (households with children), we used non-causal language to be conservative, despite the use of a causally interpretable triple difference design.

These limitations are counterbalanced by several strengths. First, we utilized data from a nationally representative, high-frequency survey, capturing variation and granularity in individuals' and households' circumstances. Second, we used a quasi-experimental triple difference approach, allowing us to build upon our and others' earlier work by exploring variation by contemporaneous economic shocks in addition to the presence of children in the household.

## Conclusion

In conclusion, our study aligns with prior work demonstrating anti-poverty benefits of the ARPA's advance CTC, which simultaneously expanded eligibility to families with no or very low income, increased maximum benefit amounts, and provided monthly in addition to annual payments. We add to this body of evidence by showing that health- and employment-related economic shocks are associated with increased household food insufficiency and that the advance CTC was associated with decreased food insufficiency, particularly among households experiencing economic shocks. Because we found a higher prevalence of both economic shocks and food insufficiency among low-income, non-Hispanic Black, and Hispanic households, permanently reinstating the advance CTC could progress equity for households that are structurally vulnerable to economic shocks by reducing household food insufficiency and related negative long-term health and education consequences for children. Members of Congress may consider the protective effect that reinstating the advance CTC could have for US households in both the short- and long-term. Legislators may also consider that reintroducing the advance CTC could increase population resilience and better protect households with children from hardship caused by health, environmental, or economic circumstances.

## Supplementary Material

qxae011_Supplementary_Data
