# Supplementary material for: Association between Child Tax Credit advance payments and food insufficiency in households experiencing economic shocks
Source: Health Aff Sch. 2024 Jan 31;2(2):qxae011. doi: 10.1093/haschl/qxae011 (PMC10986194; doi:10.1093/haschl/qxae011)
Supplement: qxae011_Supplementary_Data [file qxae011_Supplementary_Data.zip › McCann_appendix.docx]

**Supplemental Appendix:** Association between Child Tax Credit advance payments and food insufficiency in households experiencing economic shocks

**Table of contents**

**Table S1.** Statistical test of parallel trends in household food insufficiency between households with and without children and households with and without economic shocks before advance CTC implementation (January 6, 2021 through July 5, 2021)

**Table S2.** Unadjusted prevalence of economic shocks (any, health-related, and employment-related shocks) before advance CTC implementation among households with and without children and by subgroup (January 6, 2021 through July 5, 2021)

**Table S3.** Unadjusted prevalence of household food insufficiency among households with and without children, not experiencing or experiencing economic shocks, and by subgroup: before, during, and after advance CTC implementation

**Table S4.** Association between economic shocks (any, health-related, and employment-related shocks) and household food insufficiency before advance CTC implementation (January 6, 2021 through July 5, 2021)

**Table S5.** Difference-in-difference-in-difference estimates for change in household food insufficiency during implementation of the advance CTC for households with children experiencing economic shocks (N=1,126,299)

**Table S6.** Difference-in-difference-in-difference estimates for indicator of advance CTC implementation x indicator of children in household x indicator for economic shocks: alternative model specifications

**Table S7.** Difference-in-difference estimates for change in household food insufficiency during implementation of the advance CTC for households with children experiencing and not experiencing economic shocks

**Table S8.** Difference-in-difference-in-difference estimates for change in household food insufficiency during implementation of the advance CTC for households with children experiencing economic shocks, excluding time after 2022 tax filing deadline

**Table S9:** Difference-in-difference-in-difference estimates for change in household food insufficiency during implementation of the advance CTC for households with children experiencing economic shocks, low-income households

**Figure S1.** Unadjusted prevalence of economic shocks (any, health-related, and employment-related shocks) before advance CTC implementation by subgroup (January 6, 2021 through July 5, 2021)

**Methods: statistical model**

Our triple difference analysis took the form of Equation 1, where *i* indexes respondent, *h* indexes household, *s* state, and *t* time. The main exposure of interest is *CTC_t_,* a binary indicator for the period in which the advance CTC was implemented, interacted with *children_h_,* a binary indicator for whether household has children, interacted with *shock_i_*, whether the respondent was experiencing economic shocks (also adjusted for each indicator and pairwise interactions separately). Y*_hst_* is the household food insufficiency outcome. *X_iht_* represents the individual and household time-varying and demographic covariates listed in the manuscript. *S_s_* represents state fixed effects and *t_t_* represents survey wave fixed effects. *ε_ist_* represents the error term. We used household survey weights (divided by number of survey waves) to make the HPS data representative of the national population.

*Y_hst_ = CTC_t_ * children_h_ *shock_i_ + X_iht_* *+ S_s_* + *t_t_ + ε_ist_* [Equation 1]

**Table S1.** Statistical test of parallel trends in household food insufficiency between households with and without children and households with and without economic shocks before advance CTC implementation (January 6, 2021 through July 5, 2021)

|  | **Naïve model** | | **Fully-adjusted model** | |
| --- | --- | --- | --- | --- |
|  | *Change in household food insufficiency, percentage points (95% CI)* | *P-value* | *Change in household food insufficiency, percentage points (95% CI)* | *P-value* |
| **Triple difference** | | | | |
| Presence of children x presence of shocks x continuous time indicator | -0.13 (-0.63, 0.36) | 0.598 | -0.06 (-0.54, 0.42) | 0.798 |
| **Difference-in-difference** | | | | |
| Presence of children x continuous time indicator: households not experiencing shocks | 0.02 (-0.09, 0.13) | 0.716 | 0.05 (-0.05, 0.15) | 0.331 |
| Presence of children x continuous time indicator: households experiencing shocks | -0.11 (-0.60, 0.37) | 0.647 | 0.03 (-0.43, 0.49) | 0.897 |

**Abbreviations:** CI: confidence interval; CTC: Child Tax Credit

Weighted using household survey weights divided by the number of waves.

Fully adjusted model controls for age, sex at birth, race/ethnicity, education level, number of adults in household, number of children in household, annual household income, employment in last 7 days, use of unemployment insurance for spending/funds, use of Economic Impact Payments for spending/funds, Supplemental Nutrition Assistance Program participation, receipt of food aid, eviction risk, health insurance coverage, and state and wave fixed effects.

Triple difference reflects our main analysis, difference-in-difference analyses evaluate trends among households experiencing and not experiencing shocks separately.

**Table S2.** Unadjusted prevalence of economic shocks (any, health-related, and employment-related shocks) before advance CTC implementation among households with and without children and by subgroup (January 6, 2021 through July 5, 2021)

| **Sample, %**  **(95% CI)** | **Overall** | **Households without children** | **Households with children** |
| --- | --- | --- | --- |
| Any economic shocks |  |  |  |
| Overall | 10.9 (10.7-11.0) | 11.3 (11.1-11.5) | 10.3 (10.1-10.6) |
| Low-income^1^ | 20.6 (20.1-21.1) | 21.1 (20.4-21.7) | 19.9 (19.1-20.7) |
| Non-Hispanic white | 9.2 (9.0-9.3) | 10.0 (9.7-10.2) | 7.9 (7.6-8.2) |
| Non-Hispanic Black | 15.5 (14.8-16.1) | 15.6 (14.7-16.5) | 15.3 (14.4-16.2) |
| Hispanic | 14.3 (13.8-14.9) | 14.4 (13.6-15.2) | 14.3 (13.5-15.0 |
| Non-Hispanic Asian | 8.0 (7.4-8.5) | 8.7 (7.9-9.6) | 7.1 (6.3-7.9) |
| Another race/ethnicity | 13.4 (12.6-14.1) | 14.9 (13.8-15.9) | 11.6 (10.5-12.7) |
| Health-related shocks |  |  |  |
| Overall | 4.5 (4.4-4.6) | 5.0 (4.8-5.1) | 3.9 (3.8-4.1) |
| Low-income | 10.1 (9.7-10.5) | 11.1 (10.6-11.6) | 8.7 (8.1-9.2) |
| Non-Hispanic white | 4.0 (3.9-4.1) | 4.6 (4.4-4.7) | 3.2 (3.0-3.3) |
| Non-Hispanic Black | 6.4 (6.0-6.9) | 7.1 (6.4-7.7) | 5.8 (5.2-6.3) |
| Hispanic | 5.4 (5.1-5.8) | 5.6 (5.0-6.2) | 5.3 (4.8-5.7) |
| Non-Hispanic Asian | 2.2 (1.8-2.5) | 2.4 (1.9-2.9) | 2.0 (1.5-2.4) |
| Another race/ethnicity | 5.7 (5.2-6.3) | 6.9 (6.1-7.7) | 4.5 (3.8-5.1) |
| Employment-related shocks | |  |  |
| Overall | 6.4 (6.2-6.5) | 6.3 (6.2-6.5) | 6.4 (6.2-6.6) |
| Low-income | 10.5 (10.1-10.9) | 10.0 (9.5-10.5) | 11.2 (10.6-11.9) |
| Non-Hispanic white | 5.2 (5.0-5.3) | 5.4 (5.2-5.6) | 4.8 (4.5-5.0) |
| Non-Hispanic Black | 9.0 (8.5-9.5) | 8.5 (7.8-9.2) | 9.5 (8.8-10.3) |
| Hispanic | 8.9 (8.4-9.4) | 8.8 (8.1-9.4) | 9.0 (8.3-9.7) |
| Non-Hispanic Asian | 5.8 (5.3-6.3) | 6.4 (5.7-7.1) | 5.1 (4.5-5.8) |
| Another race/ethnicity | 7.6 (7.0-8.2) | 8.0 (7.2-8.8) | 7.1 (6.3-8.0) |

**Abbreviations:** CI: confidence interval; CTC: Child Tax Credit

^1^Low-income defined as annual household income reported to be <$35,000 in the prior year.

Weighted using household survey weights divided by the number of waves.

Health-related and employment-related shocks were mutually exclusive options in the Census Household Pulse Survey, and respondents could not select more than one reason for missed work. Thus, we assume that the selected option (health-related or employment-related) is the primary reason for missed work.

**Table S3.** Unadjusted prevalence of household food insufficiency among households with and without children, not experiencing or experiencing economic shocks, and by subgroup: before, during, and after advance CTC implementation

| **Sample, %**  **(95% CI)** | **Before advance CTC** | **During advance CTC** | **After advance CTC** |
| --- | --- | --- | --- |
| Full sample | | | |
| Overall (N=1,126,299) | 11.6 (11.4, 11.8) | 10.3 (10.1, 10.6) | 12.7 (12.4, 13.0) |
| Low-income (n=179,221) | 26.4 (25.8, 26.9) | 24.1 (23.4, 24.7) | 27.9 (27.1, 28.7) |
| Non-Hispanic white (n=808,207) | 8.4 (8.2, 8.5) | 7.6 (7.4, 7.8) | 9.2 (8.9, 9.5) |
| Non-Hispanic Black (n=87,960) | 20.3 (19.6, 21.1) | 18.7 (17.8, 19.6) | 23.6 (22.5, 24.6) |
| Hispanic (n=119,439) | 18.0 (17.4, 18.7) | 15.3 (14.5, 16.1) | 18.6 (17.6, 19.7) |
| Non-Hispanic Asian (n=64,661) | 5.8 (5.3, 6.4) | 4.6 (4.0, 5.3) | 5.1 (4.3, 5.8) |
| Another race/ethnicity (n=46,032) | 17.2 (16.3, 18.1) | 14.9 (13.8, 16.0) | 19.0 (17.5, 20.4) |
| Households without children, not experiencing shocks | | | |
| Overall (n=602,659) | 8.2 (8.0, 8.5) | 8.2 (8.0, 8.5) | 9.7 (9.3, 10.0) |
| Low-income (n=93,416) | 20.7 (19.9, 21.5) | 21.3 (20.4, 22.2) | 23.0 (22.0, 24.0) |
| Non-Hispanic white (n=452,931) | 6.6 (6.3, 6.8) | 6.5 (6.2, 6.7) | 7.7 (7.4, 8.1) |
| Non-Hispanic Black (n=41,753) | 14.5 (13.5, 15.5) | 15.5 (14.3, 16.7) | 17.8 (16.4, 19.2) |
| Hispanic (n=53,462) | 11.9 (11.1, 12.7) | 11.9 (10.9, 12.9) | 13.8 (12.5, 15.0) |
| Non-Hispanic Asian (n=32,230) | 4.7 (4.0, 5.4) | 3.9 (3.0, 4.7) | 3.6 (2.7, 4.5) |
| Another race/ethnicity (n=22,283) | 13.8 (12.5, 15.0) | 13.3 (11.8, 14.8) | 14.7 (12.9, 16.5) |
| Households with children, not experiencing shocks | | | |
| Overall (n=439,033) | 11.4 (11.1, 11.7) | 9.3 (9.0, 9.7) | 12.8 (12.4, 13.3) |
| Low-income (n=51,827) | 27.7 (26.6, 28.8) | 22.4 (21.2, 23.6) | 29.5 (28.0, 31.0) |
| Non-Hispanic white (n=301,990) | 7.3 (7.0, 7.6) | 6.1 (5.7, 6.4) | 8.3 (7.8, 8.7) |
| Non-Hispanic Black (n=35,867) | 21.0 (19.9, 22.1) | 18.0 (16.8, 19.3) | 25.4 (23.7, 27.1) |
| Hispanic (n=53,334) | 17.9 (16.9, 18.9) | 13.9 (12.7, 15.0) | 18.6 (17.2, 20.1) |
| Non-Hispanic Asian (n=29,001) | 4.7 (4.0, 5.4) | 4.1 (3.2, 5.1) | 5.4 (4.2, 6.6) |
| Another race/ethnicity (n=18,831) | 16.1 (14.6, 17.5) | 13.2 (11.4, 14.9) | 18.5 (16.4, 20.5) |
| Households without children, experiencing shocks | | | |
| Overall (n=52,924) | 25.6 (24.6, 26.6) | 26.2 (24.7, 27.6) | 30.6 (28.6, 32.5) |
| Low-income (n=22,448) | 34.6 (32.9, 36.3) | 34.7 (32.6, 36.9) | 38.2 (35.4, 41.1) |
| Non-Hispanic white (n=36,324) | 22.2 (21.1, 23.2) | 23.4 (21.9, 24.9) | 25.3 (23.5, 27.1) |
| Non-Hispanic Black (n=5,401) | 31.2 (27.9, 34.5) | 30.5 (26.1, 35.0) | 39.3 (34.0, 44.7) |
| Hispanic (n=6,310) | 33.6 (30.4, 36.8) | 32.9 (28.2, 37.5) | 36.2 (29.4, 43.0) |
| Non-Hispanic Asian (n=1,939) | 16.8 (12.4, 21.1) | 17.7 (12.2, 23.1) | 18.7 (11.7, 25.8) |
| Another race/ethnicity (n=2,950) | 29.5 (26.0, 33.1) | 27.6 (22.9, 32.4) | 46.0 (38.1, 53.8) |
| Households with children, experiencing shocks | | | |
| Overall (n=31,683) | 31.3 (30.0, 32.7) | 26.6 (24.9, 28.4) | 34.7 (32.4, 37.1) |
| Low-income (n=11,520) | 40.9 (38.6, 43.1) | 32.1 (29.4, 34.8) | 42.0 (38.6, 45.5) |
| Non-Hispanic white (n=16,962) | 25.1 (23.4, 26.8) | 21.6 (19.5, 23.7) | 30.4 (27.3, 33.4) |
| Non-Hispanic Black (n=4,939) | 37.9 (34.7, 41.0) | 31.4 (27.1, 35.6) | 39.1 (34.2, 44.0) |
| Hispanic (n=6,323) | 36.7 (33.8, 39.6) | 31.3 (27.6, 35.1) | 40.9 (35.1, 46.6) |
| Non-Hispanic Asian (n=1,491) | 22.3 (17.1, 27.3) | 14.1 (8.7, 19.4) | 15.9 (9.6, 22.2) |
| Another race/ethnicity (n=1,969) | 35.7 (31.0, 40.5) | 28.6 (22.2, 35.0) | 31.6 (24.5, 38.7) |

**Abbreviations**: CI: confidence interval; CTC: Child Tax Credit

^1^Low-income defined as annual household income reported to be <$35,000 in the prior year.

Weighted using household survey weights divided by the number of waves.

**Table S4.** Association between economic shocks (any, health-related, and employment-related shocks) and household food insufficiency before advance CTC implementation (January 6, 2021 through July 5, 2021)

|  | **Change in household food insufficiency,  percentage points (95% CI)** | | **P-value** |
| --- | --- | --- | --- |
| **Overall sample** | | | |
| Any economic shocks | | | |
| All households | | 7.7 (6.7, 8.6) | <0.001 |
| Households without children | | 6.7 (5.6, 7.8) | <0.001 |
| Households with children | | 8.5 (7.0, 10.0) | <0.001 |
| Health-related shocks | | | |
| All households | | 3.9 (2.6, 5.2) | <0.001 |
| Households without children | | 2.8 (1.3, 4.3) | <0.001 |
| Households with children | | 4.9 (2.8, 7.1) | <0.001 |
| Employment-related shocks | | | |
| All households | | 8.0 (6.8, 9.2) | <0.001 |
| Households without children | | 7.6 (6.1, 9.0) | <0.001 |
| Households with children | | 8.3 (6.5, 10.2) | <0.001 |
| **Low-income^1^ subgroup** | |  |  |
| Any economic shocks | |  |  |
| All households | | 7.2 (5.4, 8.9) | <0.001 |
| Households without children | | 5.8 (3.7, 8.0) | <0.001 |
| Households with children | | 7.8 (5.0, 10.7) | <0.001 |
| Health-related shocks | |  |  |
| All households | | 3.2 (1.1, 5.3) | 0.003 |
| Households without children | | 1.4 (-1.1, 3.9) | 0.274 |
| Households with children | | 4.6 (0.92, 8.3) | 0.014 |
| Employment-related shocks | |  |  |
| All households | | 8.1 (5.8, 10.4) | <0.001 |
| Households without children | | 8.1 (5.2, 11.0) | <0.001 |
| Households with children | | 7.5 (4.0, 11.1) | <0.001 |
| **Non-Hispanic white subgroup** | | | |
| Any economic shocks | |  |  |
| All households | | 6.6 (5.6, 7.6) | <0.001 |
| Households without children | | 5.8 (4.6, 7.0) | <0.001 |
| Households with children | | 7.7 (5.9, 9.5) | <0.001 |
| Health-related shocks | |  |  |
| All households | | 4.0 (2.5, 5.4) | <0.001 |
| Households without children | | 3.0 (1.3, 4.6) | <0.001 |
| Households with children | | 5.5 (2.6, 8.3) | <0.001 |
| Employment-related shocks | |  |  |
| All households | | 6.7 (5.4, 8.0) | <0.001 |
| Households without children | | 6.3 (4.7, 7.9) | <0.001 |
| Households with children | | 7.1 (4.9, 9.3) | <0.001 |

**Table S4, cont.** Association between economic shocks (any, health-related, and employment-related shocks) and household food insufficiency before advance CTC implementation (January 6, 2021 through July 5, 2021)

|  | **Change in household food insufficiency, percentage points (95% CI)** | **P-value** |
| --- | --- | --- |
| **Non-Hispanic Black subgroup** | | |
| Any economic shocks |  |  |
| All households | 5.9 (3.1, 8.8) | <0.001 |
| Households without children | 5.3 (1.4, 9.1) | 0.008 |
| Households with children | 6.0 (2.0, 10.0) | 0.003 |
| Health-related shocks |  |  |
| All households | 2.0 (-1.5, 5.5) | 0.258 |
| Households without children | -1.7 (-6.4, 3.1) | 0.496 |
| Households with children | 5.3 (0.32, 10.3) | 0.037 |
| Employment-related shocks |  |  |
| All households | 6.8 (3.2, 10.3) | <0.001 |
| Households without children | 9.5 (4.5, 14.5) | <0.001 |
| Households with children | 4.2 (-0.59, 9.1) | 0.085 |
| **Hispanic subgroup** |  |  |
| Any economic shocks |  |  |
| All households | 11.3 (8.7, 13.8) | <0.001 |
| Households without children | 11.8 (8.2, 15.3) | <0.001 |
| Households with children | 10.2 (6.7, 13.7) | <0.001 |
| Health-related shocks |  |  |
| All households | 4.7 (1.2, 8.3) | 0.009 |
| Households without children | 7.6 (2.5, 12.8) | 0.004 |
| Households with children | 1.4 (-3.3, 6.0) | 0.564 |
| Employment-related shocks |  |  |
| All households | 11.7 (8.6, 14.7) | <0.001 |
| Households without children | 9.8 (5.6, 14.0) | <0.001 |
| Households with children | 12.7 (8.4, 16.9) | <0.001 |

|  | **Change in household food insufficiency, percentage points (95% CI)** | **P-value** |
| --- | --- | --- |
| **Asian subgroup** | | |
| Any economic shocks |  |  |
| All households | 8.2 (4.7, 11.8) | <0.001 |
| Households without children | 6.6 (2.3, 10.8) | 0.003 |
| Households with children | 10.5 (4.9, 16.2) | <0.001 |
| Health-related shocks |  |  |
| All households | 11.3 (4.2, 18.3) | 0.002 |
| Households without children | 4.7 (-2.0, 11.4) | 0.170 |
| Households with children | 20.1 (7.5, 32.8) | 0.002 |
| Employment-related shocks |  |  |
| All households | 5.2 (1.6, 8.9) | 0.005 |
| Households without children | 6.1 (1.0, 11.1) | 0.018 |
| Households with children | 4.4 (-0.81, 9.5) | 0.098 |
| **Another race or ethnicity subgroup** | | |
| Any economic shocks |  |  |
| All households | 5.9 (2.4, 9.3) | 0.001 |
| Households without children | 3.1 (-1.3, 7.6) | 0.178 |
| Households with children | 8.8 (3.6, 13.9) | 0.001 |
| Health-related shocks |  |  |
| All households | 0.25 (-4.1, 4.6) | 0.909 |
| Households without children | -1.2 (-6.7, 4.3) | 0.669 |
| Households with children | 1.9 (-5.0, 8.9) | 0.586 |
| Employment-related shocks |  |  |
| All households | 8.4 (4.2, 12.6) | <0.001 |
| Households without children | 5.8 (0.31, 11.3) | 0.039 |
| Households with children | 11.0 (4.7, 17.3) | 0.001 |

**Table S4, cont**. Association between economic shocks (any, health-related, and employment-related shocks) and household food insufficiency before advance CTC implementation (January 6, 2021 through July 5, 2021)

**Abbreviations:** CI: confidence interval; CTC: Child Tax Credit

^1^Low-income defined as household income reported to be <$35,000 in the prior year.

Model controls for age, sex at birth, race/ethnicity, education level, number of adults in household, number of children in household, annual household income, employment in last 7 days, use of unemployment insurance for spending/funds, use of Economic Impact Payments for spending/funds, Supplemental Nutrition Assistance Program participation, receipt of food aid, eviction risk, health insurance coverage, and state and wave fixed effects, weighted by household survey weights divided by the number of waves.

**Table S5.** Difference-in-difference-in-difference estimates for change in household food insufficiency during implementation of the advance CTC for households with children experiencing economic shocks (N=1,126,299)

| **Characteristic** | **Change in household food insufficiency, percentage points (95% CI)** | | **P-value** |
| --- | --- | --- | --- |
| Indicator for implementation of advance CTC | | 0.8 (-0.1, 1.6) | 0.064 |
| Indicator for children in household | | 2.6 (2.1, 3.2) | <0.001 |
| Advance CTC implementation x children in household | | -1.6 (-2.1, -1.1) | <0.001 |
| Indicator for economic shocks | | 8.0 (7.0, 8.9) | <0.001 |
| Advance CTC implementation x economic shocks | | -1.4 (-3.0, 0.1) | 0.070 |
| Children present x economic shocks | | 1.0 (-0.4, 2.4) | 0.176 |
| Advance CTC implementation x children present x economic shocks | | -3.5 (-6.1, -0.9) | 0.008 |
| Sex at birth | |  |  |
| Female | | 0.1 (-0.2, 0.3) | 0.688 |
| Male | | – | – |
| Age group, y | |  |  |
| 18-24 | | – | – |
| 25-44 | | 4.0 (3.4, 4.6) | <0.001 |
| 45-64 | | 2.3 (1.7, 2.9) | <0.001 |
| Race and ethnicity | |  |  |
| Hispanic | | 2.0 (1.6, 2.4) | <0.001 |
| Non-Hispanic | |  |  |
| Asian | | -1.0 (-1.3, -0.6) | <0.001 |
| Black | | 4.4 (3.9, 4.9) | <0.001 |
| White | | – | – |
| Another race or ethnicity^3^ | | 4.1 (3.5, 4.7) | <0.001 |
| Education | |  |  |
| Less than high school | | – | – |
| High school / equivalent | | -5.6 (-6.5, -4.7) | <0.001 |
| Some college /2y degree | | -7.9 (-8.8, -7.0) | <0.001 |
| 4y degree or higher | | -11.3 (-12.2, -10.4) | <0.001 |
| Marital status | |  |  |
| Married | | -2.2 (-2.6, -1.9) | <0.001 |
| Not married | | – | – |
| Health insurance coverage | |  |  |
| Uninsured | | – | – |
| Public | | -3.2 (-3.9, -2.5) | <0.001 |
| Private | | -5.3 (-5.8, -4.9) | <0.001 |
| Respondent employed in last 7d | | -2.2 (-2.4, -1.8) | <0.001 |

**Table S5, cont.** Difference-in-difference-in-difference estimates for change in household food insufficiency during implementation of the advance CTC for households with children experiencing economic shocks (N=1,126,299)

| **Characteristic** | **Change in household food insufficiency, percentage points  (95% CI)** | **P-value** |
| --- | --- | --- |
| Report of UI benefits as spending source in last 7d | 0.6 (0.0, 1.1) | 0.044 |
| Current participation in SNAP in last 7d by anyone in household | -0.1 (-0.7, 0.5) | 0.732 |
| Receipt of food aid in last 7d by anyone in household | 10.1 (9.4, 10.9) | <0.001 |
| Report of EIP as spending source in last 7d | 1.1 (0.8, 1.5) | <0.001 |
| Report of risk for eviction in next 2m | 33.5 (32.1, 34.9) | <0.001 |
| Adults in household, no |  |  |
| 1 | – | – |
| 2 | -0.7 (-1.1, -0.3) | 0.001 |
| 3+ | 0.3 (-0.1, 0.8) | 0.133 |
| Children in household, no |  |  |
| 0 | – | – |
| 1 | -1.3 (-1.8, -0.7) | <0.001 |
| 2 | -1.2 (-1.7, -0.6) | <0.001 |
| 3+ | – | – |
| Annual household income, $ |  |  |
| <25,000 | – | – |
| 25,000-34,000 | -5.7 (-6.4, -4.9) | <0.001 |
| 35,000-49,999 | -8.6 (-9.3, -7.9) | <0.001 |
| 50,000-74,000 | -12.2 (-12.8, -11.6) | <0.001 |
| 75,000-149,000 | -14.3 (-14.9, -13.7) | <0.001 |
| 150,000+ | -13.7 (-14.4, -13.1) | <0.001 |
| Missing | -10.3 (-11.0, -9.6) | <0.001 |

**Abbreviations:** d: days; m: months; UI: unemployment insurance; SNAP: Supplemental Nutritional Assistance Program; EIP: Economic Impact Payments; CTC: Child Tax Credit

Model also included survey wave and fixed effects and a constant term, weighted by household survey weights divided by the number of waves.

**Table S6.** Difference-in-difference-in-difference estimates for indicator of advance CTC implementation x indicator of children in household x indicator for economic shocks: alternative model specifications (N=1,126,299)

| **Characteristic: Indicator for advance CTC implementation x indicator for presence of children in household x indicator for economic shocks** | **Change in household food insufficiency, percentage points (95% CI)** | **P-value** |
| --- | --- | --- |
| Main model | -3.5 (-6.1, -0.9) | 0.008 |
| Clustered SEs by state | -3.5 (-6.5, -0.5) | 0.022 |
| Varying inclusion of fixed effects |  |  |
| No fixed effects | -3.4 (-6.0, -0.9) | 0.009 |
| State fixed effects only | -3.5 (-6.1, -0.9) | 0.009 |
| Wave fixed effects only | -3.5 (-6.1, -0.9) | 0.009 |

**Abbreviations:** CI: confidence interval; CTC: Child Tax Credit

The main model controls for age, sex at birth, race/ethnicity, education level, number of adults in household, number of children in household, annual household income, employment in last 7 days, use of unemployment insurance for spending/funds, use of Economic Impact Payments for spending/funds, Supplemental Nutrition Assistance Program participation, receipt of food aid, eviction risk, health insurance coverage, and state and wave fixed effects, weighted by household survey weights divided by the number of waves.

**Table S7.** Difference-in-difference estimates for change in household food insufficiency during implementation of the advance CTC for households with children experiencing and not experiencing economic shocks

| **Characteristic: indicator for advance CTC implementation x indicator children in household** | **Change in household food insufficiency, percentage points (95% CI)** | **P-value** |
| --- | --- | --- |
| Households without economic shocks (n=1,041,692) | -1.6 (-2.1, -1.1) | <0.001 |
| Households with economic shocks (n=84,607) | -5.2 (-7.7, -2.7) | <0.001 |

**Abbreviations**: CI: confidence interval; CTC: Child Tax Credit

Model controls for age, sex at birth, race/ethnicity, education level, number of adults in household, number of children in household, annual household income, employment in last 7 days, use of unemployment insurance for spending/funds, use of Economic Impact Payments for spending/funds, Supplemental Nutrition Assistance Program participation, receipt of food aid, eviction risk, health insurance coverage, and state and wave fixed effects, weighted by household survey weights divided by the number of waves.

**Table S8.** Difference-in-difference-in-difference estimates for change in household food insufficiency during implementation of the advance CTC for households with children experiencing economic shocks, excluding time after 2022 tax filing deadline

| **Characteristic: Indicator for advance CTC implementation x indicator for presence of children in household x indicator for economic shocks** | **Change in household food insufficiency, percentage points (95% CI)** | **P-value** |
| --- | --- | --- |
| Main model (N=1,126,299) | -3.5 (-6.1, -0.9) | 0.008 |
| Time after 2022 tax filing deadline (4/15/2022) excluded (N=1,016,153) | -3.9 (-6.5, -1.2) | 0.004 |

**Table S9.** Difference-in-difference-in-difference estimates for change in household food insufficiency during implementation of the advance CTC for households with children experiencing economic shocks, low-income households

| **Characteristic: Indicator for advance CTC implementation x indicator for presence of children in household x indicator for economic shocks** | **Change in household food insufficiency, percentage points (95% CI)** | **P-value** |
| --- | --- | --- |
| Main model (N=1,126,299) | -3.5 (-6.1, -0.9) | 0.008 |
| Low-income subgroup (N=179,221) | -3.5 (-8.0, 0.1) | 0.115 |

**Figure S1.** Unadjusted prevalence of economic shocks (any, health-related, and employment-related shocks) before advance CTC implementation by subgroup (January 6, 2021 through July 5, 2021)

**Note.** Figure S1 shows the prevalence of economic shocks in households in the period before advance CTC implementation (January 6, 2021 through July 5, 2021) on the y-axis. Prevalence of any economic shocks (health-related plus employment-related shocks) is shown with blue bars. Prevalence of health-related shocks (missing work due to COVID in oneself or caregiving, or sick with another illness) is shown in dark grey. Prevalence of employment-related shocks (missing work due to COVID-related employer temporary/permanent closure, lay-offs or furlough) is shown in light grey. Prevalence of shocks is shown overall and by low-income and racial/ethnic subgroups. Error bars represent 95% confidence intervals.

**Abbreviations: CTC:** advance Child Tax Credit
