# Supplementary material for: Association between Child Tax Credit advance payments and food insufficiency in households experiencing economic shocks
Source: Health Aff Sch. 2024 Jan 31;2(2):qxae011. doi: 10.1093/haschl/qxae011 (PMC10986194; doi:10.1093/haschl/qxae011)
Supplement: qxae011_Supplementary_Data [file qxae011_Supplementary_Data.zip › ICMJE coi_disclosure_combined.docx]

| ICMJE DISCLOSURE FORM | |
| --- | --- |
| **Date:** | 10/25/2023 |
| **Your Name:** | Nicole McCann; Lorraine Dean; Allison Bovell-Ammon; Stephanie Ettinger de Cuba ; Tiffany Green ; Paul Shafer ; Julia Raifman |
| **Manuscript Title:** | Association between Child Tax Credit advance payments and food insufficiency in households experiencing economic shocks |
| **Manuscript Number (if known):** | Click or tap here to enter text. |
| In the interest of transparency, we ask you to disclose all relationships/activities/interests listed below that are related to the content of your manuscript. “Related” means any relation with for-profit or not-for-profit third parties whose interests may be affected by the content of the manuscript. Disclosure represents a commitment to transparency and does not necessarily indicate a bias. If you are in doubt about whether to list a relationship/activity/interest, it is preferable that you do so.  The author’s relationships/activities/interests should be defined broadly. For example, if your manuscript pertains to the epidemiology of hypertension, you should declare all relationships with manufacturers of antihypertensive medication, even if that medication is not mentioned in the manuscript.  In item #1 below, report all support for the work reported in this manuscript without time limit. For all other items, the time frame for disclosure is the past 36 months. | |

|  | | | **Name all entities with whom you have this relationship or indicate none (add rows as needed)** | **Specifications/Comments (e.g., if payments were made to you or to your institution)** |
| --- | --- | --- | --- | --- |
| **Time frame: Since the initial planning of the work** | | | | |
| **1** | All support for the present manuscript (e.g., funding, provision of study materials, medical writing, article processing charges, etc.)  **No time limit for this item.** | | \|  \| None \| \| --- \| --- \|  \| Commonwealth Fund (20213259) (Shafer) \| Grant to institution \| \| --- \| --- \| \| Commonwealth Fund (Raifman) \| Boston University Center for Antiracist Research \| \| None (McCann; Dean; Green; Bovell-Ammon; Ettinger de Cuba) \|  \| | |
| **Time frame: past 36 months** | | | | |
| **2** | | Grants or contracts from any entity (if not indicated in item #1 above). | \|  \| **None** \| \| --- \| --- \|  \| None (Shafer; McCann; Dean; Green; Bovell-Ammon; Ettinger de Cuba) \|  \| \| --- \| --- \| \| National Institute of Mental Health (Raifman) \| Boston University \| \| Robert Wood Johnson Foundation (Raifman) \| Boston University \| \|  \|  \| | |
| **3** | | Royalties or licenses | \|  \| **None** \| \| --- \| --- \|  \| None (Shafer; McCann; Dean; Green; Bovell-Ammon; Raifman; Ettinger de Cuba) \|  \| \| --- \| --- \| \|  \|  \| \|  \|  \| | |
| **4** | | Consulting fees | \|  \| **None** \| \| --- \| --- \|  \| None (Shafer; McCann; Dean; Green; Bovell-Ammon; Raifman; Ettinger de Cuba) \|  \| \| --- \| --- \| \|  \|  \| \|  \|  \| | |
| **5** | | Payment or honoraria for lectures, presentations, speakers bureaus, manuscript writing or educational events | \|  \| **None** \| \| --- \| --- \|  \| None (Shafer; McCann; Dean; Green; Bovell-Ammon; Raifman; Ettinger de Cuba) \|  \| \| --- \| --- \| \|  \|  \| \|  \|  \| | |
| **6** | | Payment for expert testimony | \|  \| **None** \| \| --- \| --- \|  \| None (Shafer; McCann; Dean; Green; Bovell-Ammon; Raifman; Ettinger de Cuba) \|  \| \| --- \| --- \| \|  \|  \| \|  \|  \| | |
| **7** | | Support for attending meetings and/or travel | \|  \| **None** \| \| --- \| --- \|  \| None (Shafer; McCann; Dean; Green; Bovell-Ammon; Raifman; Ettinger de Cuba) \|  \| \| --- \| --- \| \|  \|  \| \|  \|  \| | |
| **8** | | Patents planned, issued or pending | \|  \| **None** \| \| --- \| --- \|  \| None (Shafer; McCann; Dean; Green; Bovell-Ammon; Raifman; Ettinger de Cuba) \|  \| \| --- \| --- \| \|  \|  \| \|  \|  \| | |
| **9** | | Participation on a Data Safety Monitoring Board or Advisory Board | \|  \| **None** \| \| --- \| --- \|  \| None (Shafer; McCann; Dean; Green; Bovell-Ammon; Raifman; Ettinger de Cuba) \|  \| \| --- \| --- \| \|  \|  \| \|  \|  \| | |
| **10** | | Leadership or fiduciary role in other board, society, committee or advocacy group, paid or unpaid | \|  \| **None** \| \| --- \| --- \|  \| None (Shafer; McCann; Dean; Green; Bovell-Ammon; Raifman; Ettinger de Cuba) \|  \| \| --- \| --- \| \|  \|  \| \|  \|  \| | |
| **11** | | Stock or stock options | \|  \| **None** \| \| --- \| --- \|  \| None (Shafer; McCann; Dean; Green; Bovell-Ammon; Raifman; Ettinger de Cuba) \|  \| \| --- \| --- \| \|  \|  \| \|  \|  \| | |
| **12** | | Receipt of equipment, materials, drugs, medical writing, gifts or other services | \|  \| **None** \| \| --- \| --- \|  \| None (Shafer; McCann; Dean; Green; Bovell-Ammon; Raifman; Ettinger de Cuba) \|  \| \| --- \| --- \| \|  \|  \| \|  \|  \| | |
| **13** | | Other financial or non-financial interests | \|  \| **None** \| \| --- \| --- \|  \| None (Shafer; McCann; Dean; Green; Bovell-Ammon; Raifman; Ettinger de Cuba) \|  \| \| --- \| --- \| \|  \|  \| \|  \|  \| | |
|  | |  |  | |
| **Please place an “X” next to the following statement to indicate your agreement:** | | | | |
|  | | I certify that I have answered every question and have not altered the wording of any of the questions on this form. | | |
